# Surgical Management of Morel-Lavallee Lesion

**Published:** 2015-03-03

**Authors:** Nicholas Haydon, Jack Zoumaras

**Affiliations:** Department of Plastic and Reconstructive Surgery, Royal North Shore Hospital, Sydney, Australia

**Keywords:** closed internal degloving injury, Morel-Lavallee lesion, thigh trauma, wound debridement, wound management

**Figure:**
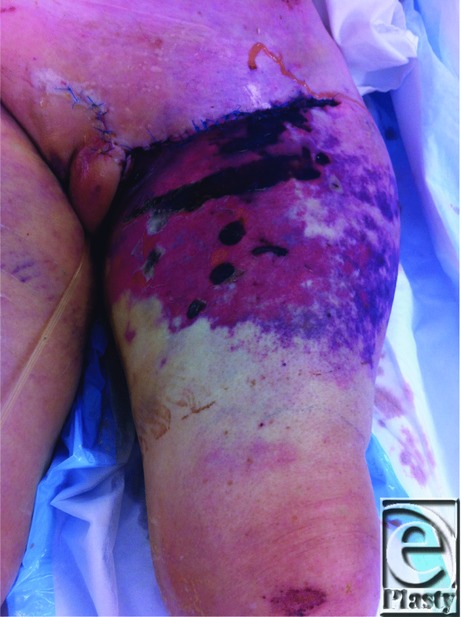

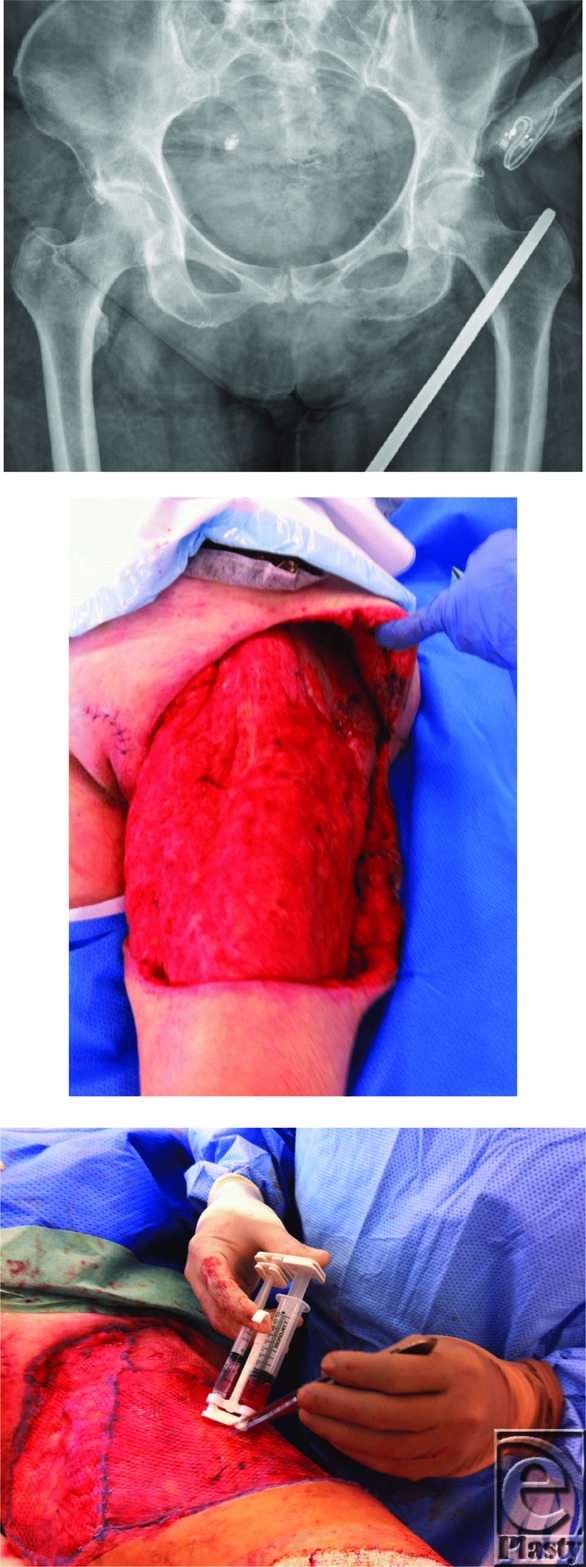


## DESCRIPTION

A 73-year-old lady sustained mutliple injuries including pelvic fractures and extensive soft tissue injuries to her left groin and thigh after being run over by a car and trailer. Closed internal degloving injury of the left thigh was diagnosed and required surgical management.

## QUESTIONS

1. **How should this patient be managed initially?**
2. **What is a Morel-Lavallee lesion?**
3. **Describe the pathophysiology of this injury?**
4. **How is this injury managed surgically?**

## DISCUSSION

The patient was transferred to a major trauma hospital with injuries to her left arm, trunk, groin, and leg for assessment and management. Initial management followed the Advanced Trauma Life Support principles. On arrival to the emergency department, her airway was patent, breathing spontaneously, with a heart rate of 70, blood pressure of 100/60, and Glasgow Coma Scale of 15. Examination revealed multiple soft tissue and skeletal injuries. Initial investigations showed Hb 90, unremarkable chest X-ray with plain trauma films demonstrating comminuted left superior and inferior pubic rami fractures and left wrist fractures. Computed tomographic scan showed the pelvic fractures and subcutaneous emphysema involving the left anterior thigh and anterior abdominal wall due to extensive thigh degloving injury.

The lady sustained significant soft tissue injury and pelvic trauma from a low-velocity motor vehicle accident. On examination, she had extensive contusions and abrasions to the left proximal thigh extending to groin and posteriorly to the buttock, as well as a perineal hematoma. Closed internal degloving injury is caused by blunt injury producing significant soft tissue injury that is associated with pelvic trauma. A Morel-Lavallee lesion is a closed internal degloving injury overlying the greater trochanter.^1^

The injury occurs when the subcutaneous tissue is sheared from the underlying fascia with avulsion of the perforating vessels. The cavity is filled with hematoma and liquefied adipose tissue. The force exerted on the superficial soft tissue also disrupts the vascular supply to the skin. This can also cause abrasions or friction burns.^2^ The area of viable skin is often difficult to determine on initial inspection.

The patient underwent exploration and washout in theater with 1.7 L of haemoserous fluid drained initially from the cavity. Thorough debridement of devascularized tissue is the mainstay of surgical management of internal degloving injuries. Often multiple debridement procedures are required to excise nonviable tissue, with vacuum-assisted closure useful for temporary coverage. (3) In some instances, the patient's condition may dictate a delay to complete debridement. Similarly, extensive open avulsion type soft tissue injuries require staged debridement. (2) Expanding hematoma in the zone of injury may further jeopardize the overlying tissue and requires immediate drainage, either percutaneously or open. (4) Delayed closure of skin flaps and/or split-thickness skin graft are used to repair the defect after adequate debridement. Undamaged but avascular skin can be reapplied to the debrided surface as a full-thickness skin graft after excising the subcutaneous fat. (2) The patient was stabilized in intensive care unit, and the left thigh skin was allowed to demarcate and then debridement and application of negative pressure dressing (vacuum-assisted closure) was performed. She required multiple procedures for further washouts and change of dressings. The defect was reconstructed with split-thickness skin graft (6/1000 inch, meshed 1:1.5) and skin flap repair with sutures and autologous platelet enriched/bovine thrombin glue.

Closed internal degloving injuries require prompt surgical assessment. Patient factors including severity of skeletal injuries direct the surgical management of the associated soft tissue injury but debridement and delayed repair remains the mainstay of treatment.
